# Alpha-synuclein and tau: teammates in neurodegeneration?

**DOI:** 10.1186/1750-1326-9-43

**Published:** 2014-10-29

**Authors:** Simon Moussaud, Daryl R Jones, Elisabeth L Moussaud-Lamodière, Marion Delenclos, Owen A Ross, Pamela J McLean

**Affiliations:** Mayo Clinic Jacksonville, 4500 San Pablo Road, Jacksonville, FL 32224 USA; Mayo Graduate School, Mayo Clinic College of Medicine, 200 1st St SW, Rochester, MN 55905 USA

**Keywords:** Tau, MAPT, Synuclein, SNCA, Oligomers, Tangles, Synucleinopathy, Tauopathy, Parkinson’s disease, Alzheimer’s disease

## Abstract

The accumulation of α-synuclein aggregates is the hallmark of Parkinson’s disease, and more generally of synucleinopathies. The accumulation of tau aggregates however is classically found in the brains of patients with dementia, and this type of neuropathological feature specifically defines the tauopathies. Nevertheless, in numerous cases α-synuclein positive inclusions are also described in tauopathies and *vice versa*, suggesting a co-existence or crosstalk of these proteinopathies. Interestingly, α-synuclein and tau share striking common characteristics suggesting that they may work in concord. Tau and α-synuclein are both partially unfolded proteins that can form toxic oligomers and abnormal intracellular aggregates under pathological conditions. Furthermore, mutations in either are responsible for severe dominant familial neurodegeneration. Moreover, tau and α-synuclein appear to promote the fibrillization and solubility of each other *in vitro* and *in vivo*. This suggests that interactions between tau and α-synuclein form a deleterious feed-forward loop essential for the development and spreading of neurodegeneration. Here, we review the recent literature with respect to elucidating the possible links between α-synuclein and tau.

## Introduction

Age-related neurodegenerative disorders like Alzheimer’s disease (AD) and Parkinson’s disease (PD) take an overwhelming toll on individuals and society [1]. AD and PD are the two most frequent neurodegenerative diseases (http://www.who.org). To date, PD and AD remain incurable and only very limited palliative treatment options exist [2]. The etiology of PD and AD is not fully understood, but appears to involve a complex combination of environmental and genetic factors [3].

**Figure 1:**
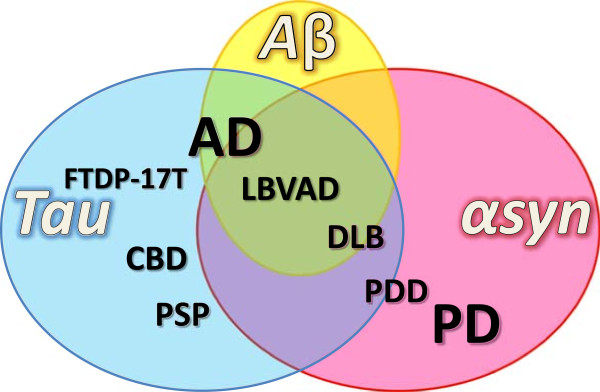
**Overlap of proteinopathies.** In numerous neurodegenerative disorders, amyloid deposits composed of α-synuclein protein (red circle), tau protein (blue circle) and Aβ peptide (yellow circle) are found. Histopathological classification of neurodegenerative diseases is based on the nature and localization of these deposits in the nervous system. The pathologies are not hermetically isolated categories but form a continuum and concomitance of αsyn and tau pathology is not rare. αSyn pathology (or synucleinopathy) is not restricted to PD but is a feature of several dementing disorders such as PDD, DLB, and frequently occurs in AD where it contributes to secondary symptoms. By contrast tauopathy is repeatedly observed in numerous disorders primarily classified as synucleinopathies and may contribute to clinical heterogeneity.

**Figure 2:**
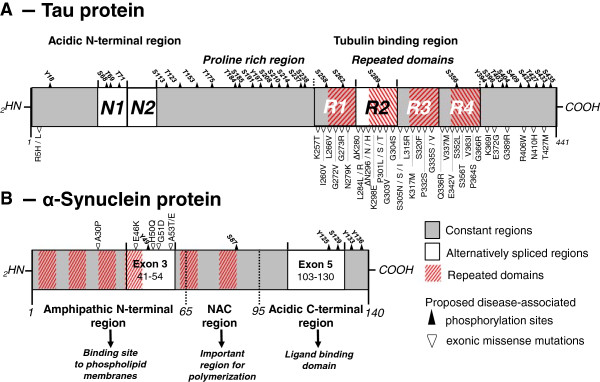
**Schematic representation of tau and α-synuclein proteins. A**- Alternative splicing of the N1, N1 + N2 and R2 regions (white) yields in 6 different tau isoforms referred to as 0N3R (=tau23 or tau-352), 0N4R (=tau24 or tau-383), 1N3R (=tau37 or tau-381), 1N4R (=tau46 or tau-412), 2N3R (=tau39 or tau-410) and 2N4R (=tau40 or tau-441). Tau has an acidic N-terminus and a tubulin binding region where the vast majority of the exonic (▽) and intronic (not depicted here) disease-associated mutations are found. **B**- αSyn is a 14.5 kDa protein divided into 3 major regions; the amphipathic N-terminus, the hydrophobic Non-Amyloid Component (NAC) domain, and the acidic C-terminus. Pathogenic missense mutations described to date (▽) are located in the N-terminal region, whereas most disease-related phosphorylation sites (▲) are localized to the C-terminal region of the protein.

**Figure 3:**
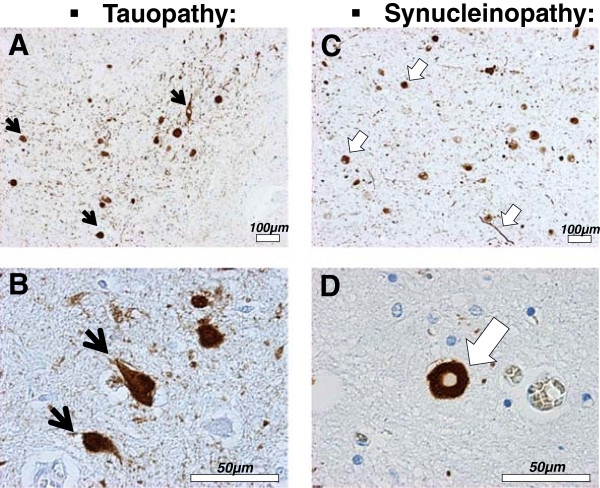
**Tau and α-synuclein pathologies. A to D-** Histological sections from the *amygdala* of DLB patients immunostained with an antibody against phosphorylated tau (PHF-1, Abcam, #ab66275) **(A and B)** or an antibody against phosphorylated αsyn (pSyn#64, Wako, #015-25191) **(C and D)**. Abnormal proteinaceous inclusions of phosphorylated tau protein, called neurofibrillary tangles **(A and B,**  **)**, and of αsyn protein, called Lewy bodies and neurites **(C and D,**  **)** are often found in neurons of the *amygdala* in DLB patients.

Interestingly, at the molecular level, protein misfolding, accumulation, aggregation and subsequently the formation of amyloid deposits are common features in many neurological disorders including AD and PD. Thus neurodegenerative diseases are sometimes referred to as proteinopathies [4]. The existence of a common mechanism suggests that neurodegenerative disorders likely share a common trigger and that the nature of the pathology is determined by the type of the aggregated protein and the localization of the cell affected (Figures 1, 2 and 3).

PD is pathologically characterized by the presence of Lewy bodies in the subcortical regions of the brain, which are composed of aggregated and phosphorylated alpha-synuclein protein (αsyn) (Figures 2 and 3) [5]. Hence PD belongs to a cluster of neurodegenerative disorders called synucleinopathies, which also includes Parkinson’s disease with dementia (PDD), dementia with Lewy bodies (DLB) and multiple system atrophy (MSA) [6, 7] (Figure 1). AD can be classified as a tauopathy (as well as an amyloidopathy); a class of disorders with intracellular inclusions composed of hyperphosphorylated and aggregated tau protein in the form of neurofibrillary tangles or Pick’s bodies (Figures 2 and 3) [8]. Tauopathies also include frontotemporal dementia with parkinsonism linked to tau mutations on chromosome 17 (FTDP-17 T), Pick’s disease (PiD), progressive supranuclear palsy (PSP) and corticobasal degeneration (CBD) (Figure 1).

The concept of the existence of a continuum between pure synucleinopathies and tauopathies has emerged and is supported by clinical observations of a high comorbidity and overlap between neurodegenerative disorders (Figure 1), in particular between dementia and parkinsonism [9]. In this continuum theory, two proteins are central: tau and αsyn. Both form abnormal intracellular inclusions, and mutations in either are sufficient to cause neurodegeneration. Recently new data has emerged which suggests that αsyn and tau may interact, and that this interaction is essential for the development and spreading of neurodegeneration. In the present manuscript we discuss the recent data in line with this paradigm.

## Co-occurrence of tauopathies and synucleinopathies

There are many exceptions to the classical view that αsyn and tau pathology are the hallmarks of PD and AD [10], the obvious being that incidental tauopathy or synucleinopathy is sometimes observed in asymptomatic patients [11–14]. Furthermore tauopathies and synucleinopathies are not restricted to pure AD and PD respectively, but rather encompass a variety of other disorders in which co-occurrence of tau and αsyn inclusions is frequent such as in PDD, DLB, Lewy body variant of AD (LBVAD), Guam-Parkinson-ALS dementia complex [15, 16] and even Down’s syndrome [17] (Figure 1). Additionally, there is considerable crosstalk and comorbidity between PD and AD. For instance, PD patients are at increased risk of developing dementia [10, 18–21] and more than half of AD patients have Lewy bodies at autopsy, particularly in the amygdala [17, 22–24], with the presence of Lewy bodies correlating with faster and more aggressive pathology [25]. In sporadic PD, neurofibrillary tangles have been repeatedly described over the past century [26–29] and synaptic-enriched fractions of AD, PD, and DLB brains have been shown to contain high levels of S396 phospho-tau and phospho-αsyn [30]. Interestingly, dementia and pronounced tau pathology have been described in familial cases of parkinsonism linked to αsyn gene (*SNCA*) mutations [31–34]. In addition, in other familial forms of parkinsonism linked to *PARKIN* or *LRRK2* gene mutations, the inconsistent accumulation of tau, αsyn, neither, or both proteins has been observed [10, 35].

In PD and PDD cases with tauopathy, phospho-tau is restricted to striatal tissues and dopaminergic neurons [36, 37] and some studies even co-localized tau and αsyn in the same aggregates. For instance in PD and DLB cases, phospho-tau and αsyn were sometimes found together in neurofibrillary tangles, Lewy bodies and neurites [38, 39]. In one study using mass spectrometry, tau was found as a component of Lewy bodies in addition to tubulin and other cytoskeletal proteins [40]. However at the molecular level, αsyn and tau were shown to segregate into different fibrillar species within one single aggregate [38].

## *SNCA*and *MAPT*in genetic studies

It is fascinating to observe that familial cases carrying mutations in the microtubule-associated protein tau (*MAPT*) or *SNCA* genes can phenotypically present with a combination of both parkinsonism and dementia. For instance, familial forms of parkinsonism due to αsyn pathogenic substitutions (A30P, E46K, H50Q, G51D or A53T) or due to the duplication or triplication of the wild-type (wt) *SNCA* gene commonly present with additional atypical clinical signs such as hallucinations, cognitive impairment, and dementia [32, 35, 41–47].

Mutations in the *MAPT* gene also cause a variety of neurodegenerative phenotypes including parkinsonism. Pathogenicity of *MAPT* splice-site and missense mutations such as G272V, N279K, P301L, V337M and R406W were first reported to cause FTDP-17 T in 1998 (Figure 2) [48–52] followed by the description of many other intronic and exonic mutations (for reviews [53–56]). While most of the mutations such as P301L and N279K primarily cause familial FTD [50, 57], other phenotypes such as CBD [58, 59], PSP [60] and variable extent of parkinsonism have been observed in some patients and families with *MAPT* mutations. Whereas the S305N mutation provokes FTD with minimal parkinsonism [61], the K369I mutation is responsible for L-DOPA sensitive parkinsonism [62] and the deltaN296 mutation is related to familial atypical PSP [63]. Surprisingly, even single *MAPT* mutations cause considerable phenotypic heterogeneity even within a single family, with a diverse combination of symptoms and age of onset [64]. This apparent randomization of the symptoms raises some questions about the exact role and specificity of tau in neurodegeneration and suggests that tau is a trigger for diverse neurodegenerative cascades. Interestingly, no consistent synuclein pathology has been reported in FTDP-17 T patients. The presence of tauopathy combined with the absence of Lewy bodies in FTDP-17 T and post-encephalitic parkinsonism cases suggests that tau alone is sufficient to provoke severe neurodegeneration leading to parkinsonism [65, 66]. However, the absence of macroscopic Lewy bodies does not exclude a role for αsyn in the form of discrete oligomers.

Recently, large-scale unbiased population-based genotyping studies have attempted to associate disease susceptibility with common genetic variants. For PD, genome-wide association studies have identified at least 24 loci so far [67]. Among them, regions encompassing the *GAK*, *HLA-DRB5, SNCA*, *LRRK2* and *MAPT* genes were the most significant hits [68–70]. The observation that common genetic variation in the *SNCA* and *MAPT* loci associates with susceptibility to disease supports a role for these genes in not only rare familial cases but also in sporadic PD. Common genetic variations at the *MAPT* locus can on the whole be divided in two major haplogroups named H1 and H2 that arose due to an ancient ~900 kb chromosome inversion. In comparison to H1, the H2 haplotype has been shown to correlate with lower expression of tau protein and to have a protective effect in neurodegeneration [71]. The *MAPT* H1 haplotype is therefore considered a genetic risk factor for a myriad of neurodegenerative disorders, including both pure tauopathies (PSP [60] and CBD [72–74]) and synucleinopathies (PD [75], PDD [76, 77] and MSA [78]). However, the H1 haplotype is very polymorphic and the specific genetic variants that associate with risk for each disorder are still not clearly defined.

In DLB, although no significant association of the *MAPT* locus with disease susceptibility was found in a recent genome-wide association study [79], correlation between H1 haplotype and the degree of synuclein pathology in the brainstem was observed in a small neuropathological study [80]. The *SNCA* single nucleotide polymorphism (SNP) rs2572324 has been correlated with the extent of neocortical Lewy body and neurofibrillary pathology [81]. These observations indicate that tau and αsyn may influence their reciprocal aggregation and suggest that their interaction is a determining factor for the development of dementia and parkinsonism. Other genetic/epidemiological studies have also indirectly linked *MAPT*/tau with PD. For instance, a SNP located within the *RIT2* gene, was recently nominated through a meta-analysis of genome-wide association studies. GTP-binding protein Rit2 binds to calmodulin 1 (phosphorylase kinase, delta), which also binds to both tau and αsyn [68]. Increased PD susceptibility was also associated with two SNPs in the *GSK3β* gene, an established tau kinase [82], although these results could not be confirmed in a subsequent study or within the genome-wide association efforts [83]. Interestingly some epidemiological studies have also tried to determine if there is an evidence of an epistatic interaction between genetic variation of the *SNCA* and *MAPT* loci. An additive or even multiplicative effect between polymorphisms in *SNCA* and *MAPT* would be expected if both genes interact within the same pathogenic pathway. One study did suggest a synergistic increase in the susceptibility of developing dementia in patients with PD when a *SNCA* risk allele was analyzed with *MAPT* H1/H2 inversion polymorphism [76]. Conversely, no synergistic effect for *SNCA* and *MAPT* (or *LRRK2*) polymorphisms were found to increase PD susceptibility in two other epidemiological studies [84, 85] and one meta-analysis [86].

## Tau and α-synuclein *in vivo*models

### Toxin-based rodent models

Prior to the discovery of the genetic forms of disease, i.e. mutations of *MAPT* and *SNCA*, toxin-based rodent models characterized *in vivo* parkinsonism research. The discovery that dopaminergic mid-brain neurons are especially sensitive to oxidative stress inducers such as 1-methyl-4-phenyl-1,2,3,6-tetrahydropyridine (MPTP), 6-hydroxydopamine (6-OHDA), rotenone, and paraquat resulted in the creation of toxin-based rodent models to study parkinsonian phenotype *in vivo* (for review [87]). Interestingly, some studies also reported the accumulation of hyperphosphorylated tau in rodents after the systemic delivery of rotenone, paraquat, and MPTP, but not maneb [88–90]. In rotenone treated rats, Hoglinger and colleagues described fibrillar structures composed of 15 nm straight filaments positive for phospho-tau, thioflavin S, nitrosamine, and ubiquitin [89]. Insoluble and phosphorylated tau has been described in mice treated with MPTP [88] and Duka and colleagues demonstrated that MPTP treatment induces tau hyperphosphorylation on the S396 and S404 residues via GSK3β kinase in wt but not in αsyn knock-out (KO) mice [91]. Later, Qureshi and Paudel confirmed that αsyn presence is required for the MPTP-induced phosphorylation of tau at S214, S262, S396 and S404 residues and identified GSK3β and PKA as the responsible kinases [92]. However, a connection between tauopathy and synucleinopathy has not been consistently observed in these PD toxin-based models. In an interesting study by Morris and colleagues, a reduction of tau expression did not prevent 6-OHDA neurotoxicity [93]. These apparently contradictory results demonstrate that the interplay between tau and αsyn is complex.

### αSyn and tau viral overexpression in rodents

The effect of targeted human αsyn and tau protein expression has been investigated using viral vectors-based models. In these models, co-occurrence of tau and αsyn pathologies has also been observed. For instance, in rats, αsyn overexpression induced by stereotaxic injection of lentivirus increases phospho-tau levels [94]. On the contrary, rats transduced with tau and mutant P301L tau display an increase of αsyn and phospho-αsyn levels [95]. In another study using adeno-associated vectors for gene transfer into the *substantia nigra* of rats, overexpression of human wt and P301L tau, but not αsyn, provoked dopaminergic neurodegeneration, reduced striatal dopamine content, and motor deficit as measured by amphetamine-stimulated rotational behavior [96]. In this study behavioral dysfunction preceded the formation of neurofibrillary tangles suggesting that mature neurofibrillary tangles are not required for tau-induced disruption of dopaminergic transmission [96].

### Tau and αsyn genetic mouse models

Numerous genetically modified mice that overexpress the human tau and/or αsyn proteins have been generated and are used to model specific aspects of the human diseases. Interestingly, tau transgenic models not only develop cognitive changes but also motor dysfunction. Mice overexpressing the mutant K369I tau develop L-DOPA sensitive parkinsonism [62], and overexpression of the pathogenic P301L and P301S forms of tau in mice provoke severe motor dysfunctions that recapitulate some of their effects in humans [97, 98]. In the P301L tau overexpressing mouse line, inhibition of tau hyperphosphorylation by treatment with a non-specific protein kinase inhibitor also prevents the motor impairments suggesting that tau could be a target in degenerative movement disorders [99].

Likewise, cognitive deficits and tauopathy have been observed in αsyn-overexpressing PD models [100–103]. Interestingly different extents of tauopathy and cognitive impairment were observed depending on the promoter type used for αsyn overexpression and the species of αsyn expressed. The presence of hyperphosphorylated tau was clearly identified in a wt αsyn overexpressing transgenic mouse line using the PDGF-β promoter at 11 months of age [101, 102]. Nonetheless, no tauopathy was observed at 18 months in a transgenic line overexpressing wt αsyn under the prion promoter unless this line was crossed with a P301L tau mouse [100]. Interestingly, in two other lines also using the prion promoter to overexpress the A53T and E46K mutant forms of αsyn, abundant tau inclusions were observed without the need of crossing with the P301L tau expressing line [100, 104, 105]. The E46K line was reported to have more tau threads than the A53T αsyn overexpressing transgenic line suggesting mutation-induced differences [104]. In these mice, tauopathy was restricted to areas with abundant αsyn expression and initiated simultaneously with synucleinopathy in an age-dependent fashion, although not always localized within the same cells [100]. In the PDGF-β-wt-αsyn mice, hyperphosphorylated tau was primarily found in the brainstem and in the striatum [101, 102]. Kaul and colleagues correlated phospho-tau occurrence with the activation of ERK and JNK but not of GSK3β and p38MAPK kinases, whereas Haggerty and colleagues noted a match between the presence of phospho-tau and phospho-GSK3β. Similarly, in the prion promoter driven A53T αsyn mice, as well as in patients harboring the A53T mutation, hyperphosphorylated and non-soluble tau accumulated in the striatum and was correlated with increased levels of phospho-GSK3β [105, 106]. In contrast, in a Thy-1 promoter driven A30P αsyn overexpressing transgenic mouse line, hyperphosphorylated and non-soluble tau accumulated in the brainstem in correlation with increased phospho-JNK level [107]. Jointly, these observations suggest the existence of complex region- and time-dependent interactions between kinases, αsyn and tau.

Some groups also crossed different transgenic mouse models to trigger the co-occurrence of tauopathy and synucleinopathy with the final aim to better model complex human disorders like DLB. For instance, a quadruple transgenic mouse line has been generated by crossing a triple transgenic mouse that overexpresses human AD-causing M146V presenilin-1, APP Swedish mutation, and the FTDP-17 T-causing P301L tau with a transgenic mouse that overexpresses human PD-causing A53T αsyn [108]. Co-overexpression of these pathogenic proteins had a strong synergistic effect on neurodegeneration, protein aggregation, and on cognitive and motor deficits. In contrast, crossing of a Thy1 promoter driven human wt αsyn overexpressing mouse line with a tau KO or tau conditional KO mouse did not prevent neurotoxicity indicating that αsyn also acts independently from tau [93].

Several tau- and αsyn- deficient mouse lines have been generated to determine if any particular phenotype or resistance to neurodegeneration might be present. Generally, tau- or αsyn-deficient mice are viable with only minor phenotypic differences [109–112]. Remarkably, cognitive alterations were observed in an α- and γ-synuclein double KO mouse line suggesting that both proteins have a compensatory role on cognition [103]. Conversely, in aged tau-deficient animals minor motor deficits were observed in correlation with an iron accumulation and loss of dopaminergic neurons in the *substantia nigra* [113], but could not be reproduced in a subsequent study [112]. Together these data demonstrate that the absence of both proteins does not appear to have observable significant impact, perhaps due to compensatory mechanisms, whereas their overexpression, in particular in their mutated forms, recapitulates some aspects of the human pathologies. This is in line with a gain of toxic function mechanism and validates therapeutic strategies aimed at clearing tau and/or αsyn for parkinsonism and/or dementia.

### Non-vertebrate models

In addition to rodents, non-vertebrate αsyn and/or tau transgenic *in vivo* models have been developed. These models work surprisingly well and present many practical aspects [114, 115]. For example, in *Caenorhabditis elegans*, expression of the human αsyn or tau protein in neurons recapitulates key features of the human diseases such as motor deficits and neuronal and dendritic loss [116]. Human αsyn expression in *Drosophila melanogaster* also induces neurotoxicity as well as L-DOPA-sensitive motor deficits and formation of αsyn-positive fibrils [117]. Remarkably, whereas αsyn expression provokes the formation of Lewy body-like aggregates in *D. melanogaster* but not in *C. elegans,* tau expression conversely leads to the formation of insoluble inclusions in *C. elegans* but not in *D. melanogaster* [115, 116]. Nonetheless, both proteins are neurotoxic in both models demonstrating that the formation of protein aggregates is fundamentally unnecessary for toxicity. In line with this idea, dopaminergic neurons in αsyn expressing *D. melanogaster* were rescued without suppressing the presence of αsyn inclusions by co-expression of the Hsp70 chaperone [118]. More recently, co-expression of tau and αsyn in *D. melanogaster* has been shown to induce motor dysfunction, dopaminergic denervation, cytotoxicity, formation of abnormal ubiquitin positive inclusions, axonal transport disruption, and cytoskeletal and synaptic disorganization [119]. Tau affected dopaminergic cell count only when co-expressed with αsyn, demonstrating the existence of a synergistic deleterious effect between tau and αsyn once more. However, in contrast to what was observed in rodent models, the mechanism of toxicity in *D. melanogaster* was linked to severe cytoskeletal and axonal disorganization and subsequent synaptic alterations rather than αsyn–promoted tau hyperphosphorylation [119].

Interesting findings have also been made in yeast models. In *Saccharomyces cerevisiae*, overexpressing human tau does not induce significant toxicity [120, 121]. However, co-expression of tau with human αsyn leads to greater toxicity than αsyn expression alone, and also leads to the formation of insoluble hyperphosphorylated tau and αsyn aggregates. The synergistic deleterious effects were increased by expression of A53T αsyn or P301L tau instead of their wt forms [120, 122]. Finally, in these models, yeast orthologs of the cyclin-dependent kinase 5 and GSK3β kinases were shown to be involved in the phosphorylation of tau and in the αsyn plus tau induced-toxicity [120, 122].

In various transgenic or toxin-induced PD models ranging from mice to yeast, the existence of a deleterious and emulative action between tau and αsyn has been repetitively shown. This corroborates what has been observed in humans (see Co-occurrence of tauopathies and synucleinopathies and *SNCA* and *MAPT* in genetic studies sections) and reinforces the idea that the interplay between αsyn and tau are pivotal in the neurodegenerative process. Nonetheless, results from these *in vivo* models have to be interpreted with caution. For instance, there is no ortholog gene of the human *SNCA* in the fly, worm or yeast, whereas in rodents the endogenous wt αsyn carries the A53T mutation without deleterious effect [123, 124]. Moreover, in rodents, tau hyperphosphorylation can occur in instances of hibernation or starvation, making this pathological hallmark difficult to interpret [125, 126].

## Tau and α-synuclein in molecular studies

The fact that αsyn and tau can physically interact with each other was demonstrated by Jensen and colleagues in 1999. In this pioneering study, tau protein from brain lysates was pulled down by αsyn affinity chromatography. The authors also noted a strong effect of ionic strength on the binding, indicating the implication of salt bridges in the interaction [127]. Moreover, in line with an interaction under physiological conditions, a binding IC50 value of 50pM was calculated between tau and αsyn using plasmon surface resonance and a radioactive binding assay [127]. At the cellular level, tau and αsyn were co-localized in the same cellular compartments and in particular in axons [127]. This was confirmed by Förster resonance energy transfer (FRET) in more recent studies [128, 129].

### Docking sites and effects of mutations on tau – αsyn interactions

Several studies have tried to identify the exact regions and the critical amino acid residues by which tau and αsyn interact. Using protein fragmentation and recombinant peptides, Jensen and colleagues found that the interacting domains are localized to the C-terminus of αsyn (55 to 140) and the microtubule binding region of tau (192 to 383) [127]. Accordingly two subsequent studies found that the N-terminal (1 to 153) and C-terminal (352 to 441) fragments of tau do not interact with αsyn [129, 130]. The question of the role of phosphorylation and disease-related mutations in the tau and αsyn interaction has also been addressed and investigated *in vitro*. Phosphorylation of the serine 214 residue of tau was identified to increase αsyn binding [92]. In contrast, phospho-mimic/dead mutations at the serine 129 residue of αsyn had no effect [129]. No effect of A30P and A53T αsyn disease-related mutations was initially reported [127], but later in a study using FRET, the αsyn mutation A30P, but not the A53T and E46K, was shown to reduce association of αsyn with tau [129]. Conversely, in a third study using co-immunoprecipitation, αsyn mutations A30P, A53T, E46K but not E83P increased binding with tau [92], E83P being an artificial mutation in the NAC domain that blocks αsyn aggregation [131]. On the contrary, the P301L tau mutation was found to reduce interaction with αsyn [130]. Consequently the exact role of the disease-related mutations on tau and αsyn interaction still needs to be clarified especially since *in vivo* observations suggest that they may play a role.

### Tau – αsyn – kinases

**Figure 4:**
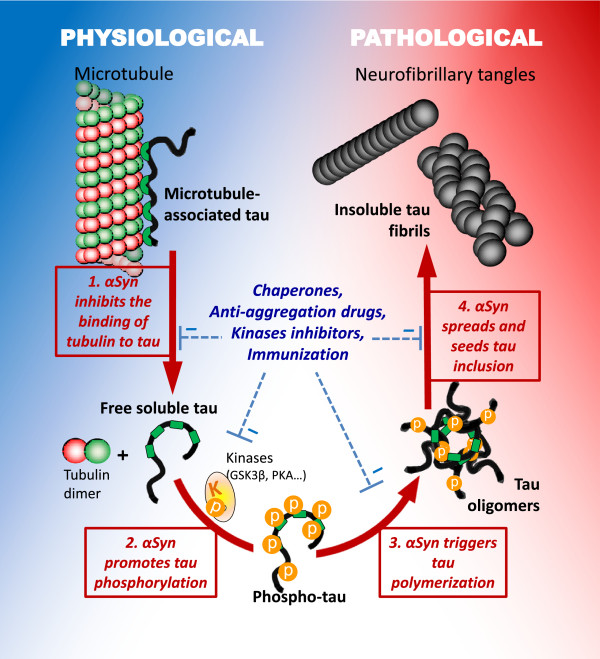
**Putative pathways of deleterious tau and α-synuclein interactions.** Interaction of tau and αsyn may promote pathogenesis via distinct mechanisms. 1. αSyn may block the normal interaction between tau and tubulin by directly binding to tau and tubulin and thereby interfering with tau physiological function. 2. αSyn could recruit kinases and promote the hyperphosphorylation of tau. 3. αSyn may also catalyze tau polymerization and trigger the formation of tau/αsyn co-oligomers. 4. Finally, αsyn oligomers or fibrils may seed tau fibrillization and thereby initiate and propagate tauopathy.

The promotion of tau hyperphosphorylation by αsyn has been demonstrated in several studies and could be a mechanism that explains how αsyn triggers tauopathy (Figure 4). An *in vitro* study showed that αsyn promotes tau phosphorylation at S262 and S356 residues via PKA [127]. Later, another tau kinase, GSK3β, was found to be recruited and activated in an αsyn-dependent manner and provoke tau hyperphosphorylation at T181, S396, and S404 residues [88, 91, 120, 132]. This effect seems to be the result of both an increase GSK3β kinase activity [91, 105] and the formation of a tripartite GSK3β-αsyn-tau complex with tau binding to the acidic C-terminus of αsyn, and GSK3β to the NAC and KTEGV domains of αsyn [132]. However, it has also been shown that similar to αsyn, β- and γ-synuclein can also induce GSK3β autophosphorylation and that β-synuclein could even promote tau phosphorylation, questioning the specificity of this mechanism and its physiological relevance [132]. Nonetheless other facts reinforce the idea of a link between GSK3β, tau and αsyn. For instance, in a cellular MPTP model, GSK3β inhibition with lithium or TDZD-8 was able to decrease tau phosphorylation but also αsyn accumulation and cell death [91]. However GSK3β is not the only kinase that links αsyn with hyperphosphorylated tau. Indeed, activation of ERK and JNK, that also phosphorylate tau at S396 and S404 residues, correlate with the presence of phospho-tau in αsyn overexpressing transgenic mouse models [102, 106, 107]. In addition, tau phosphorylation at S262 and S356 residues by PKA is exacerbated by αsyn *in vitro* [127]. Following this, PKA was identified as the responsible kinase for the αsyn-dependent phosphorylation of tau at S262 residue after MPTP treatment in cells [92]. Interestingly, PKA does not phosphorylate tau at S396 and S404 residues, whereas GSK3β does not phosphorylate tau at S262 residue suggesting that both kinases probably have an additive role in the induction of tauopathy by αsyn (Figure 4). Recently, tau has been identified as a putative substrate for the PD-related kinase LRRK2 [133, 134] and genetic correction of the PD-related LRRK2 G2019S mutation in human induced pluripotent stem cells resulted in a decreased tau and αsyn expression [135], linking tau once more to PD.

### Tau – αsyn – tubulin

αSyn may trigger tauopathy through the destabilization of the tau-tubulin interaction, which results in both tau aggregation and cytoskeleton disorganization (Figure 4). Interestingly, the 14-3-3 protein that shares some homology with αsyn [136] has also been found to bind to tau and cause tubulin instability [137]. Tau binding to tubulin is reduced by both direct competition with αsyn and indirectly by αsyn-promoted tau hyperphosphorylation [127]. Nevertheless the overall role of αsyn on cytoskeleton modeling is difficult to interpret. Indeed, similar to tau, wt but not mutant αsyn is capable of binding to tubulin and promoting tubulin polymerization [138–140]. Overexpression of A30P, A53T, E46K but not E83P mutated αsyn has been shown to decrease microtubule stability and promote phosphorylation of tau at the S262 residue by PKA [92]. Involvement of αsyn in cytoskeleton stability was also demonstrated by the disruptive effect of treatment with microtubule-destabilizing agents such as colchicine, nocodazole and vinblastine on the tubulin-αsyn interaction [129, 141]. Interestingly it was recently shown that seeds of αsyn dose-dependently reduce tau-promoted microtubule assembly, whereas αsyn oligomers impair microtubule-kinesin interplay [142].

### Co-aggregation and seeding

In the last few years, effort has been directed at unravelling the mechanisms by which neurodegeneration progresses in the brain. In PD and AD, neurodegeneration and protein aggregation overlap with each other and seem to follow a preset path giving the impression of spreading [3]. Moreover, the existence of a prion-like cell-to-cell propagation mechanism has been suggested by the unexpected *post mortem* observation that αsyn aggregates spread to healthy transplanted neurons in PD patients [143, 144]. Consistently, infection of healthy cells by a seeding mechanism similar to the self-templating activity of prions has been shown *in vitro* and *in vivo* using polymerized αsyn and tau [145–154]. Moreover, αsyn and tau have been shown to be excreted from cells via non-conventional mechanisms and are found in exosomes [155, 156].

In this perspective, a major difference between tau and αsyn is that αsyn is prone to self-aggregate, whereas tau cannot aggregate by itself and requires an inducing agent [157]. This has raised the question of whether αsyn could initiate tau aggregation; indeed, in 2003, Giasson and colleagues demonstrated that αsyn and tau promote each other’s aggregation *in vitro*. Whereas the six alternative spliced variants of tau were able to aggregate in the presence of full length wt αsyn, tau aggregation was neither promoted by the delta71-82 truncated form of αsyn, nor by β-synuclein, nor by amyloidogenic Aβ peptide [100]. These observations demonstrate that tau accelerates αsyn polymerization and that αsyn can act as an inducing agent of tau polymerization through its hydrophobic NAC region. Interestingly, mutant A53T αsyn was shown to have increased tau fibrillization properties *in vitro* when compared to wt αsyn [33]. Conversely, tau expression enhanced toxicity and secretion, and changed the pattern of αsyn aggregation by promoting the formation of smaller inclusions in cellular models [128].

Using the fluorescent intensity distribution analysis technique (FIDA), Nübling and colleagues have shown that tau and αsyn can form co-oligomers and that co-aggregation happens even at nanomolar concentrations but only in the presence of a cationic aggregation inducer such as Al^3+^ and Fe^3+^ or DMSO [158]. Moreover, tau phosphorylation by GSK3β strongly enhanced the formation of mixed oligomers [158]. However electron microscopy revealed that co-incubation of monomeric tau and αsyn mainly leads to the formation of homopolymeric fibrils [100]. This is consistent with the observations made in DLB cases [38] and suggests that αsyn and tau predominantly interact with each other at the monomeric and oligomeric stages. More recently, a series of studies tried to reproduce these *in vitro* findings *in vivo* by demonstrating that exogenous αsyn can be taken up by neurons and induce the formation of intracellular Lewy body-like structures [159, 160] and also hyperphosphorylated tau aggregates [151, 161]. For instance extracellular treatment with polymerized recombinant human αsyn induced the formation of insoluble phosphorylated tau in cellular models [104, 161]. Counterintuitively, wt αsyn fibrils were more efficient than E46K αsyn fibrils at cross-seeding tau [104].

## Conclusions and future directions

The overlap and numerous similarities between synucleinopathies and tauopathies suggest that therapeutic strategies that target common processes of tau and αsyn aggregation could benefit patients across a spectrum of neurodegenerative disorders, and may be particularly relevant for the treatment of secondary symptoms such as cognitive impairment in PD or secondary parkinsonism in dementia. In the present review we have compiled data from the literature linking tau and αsyn. The repeated *in vitro* and *in vivo* observations that tau and αsyn interact highly suggest that αsyn and tau play as teammates, however how this interaction occurs and affects neurodegenerative processes is still not fully elucidated and several scenarios are possible (Figure 4).

αSyn was initially shown to bind to tau and interfere with the normal interaction between tau and tubulin [127]. The disruption of the normal physiological interaction between tau and tubulin for a pathological interaction between tau and αsyn could explain why αsyn and tau interaction seems to be deleterious. However, more recent data suggest that the role of tau and αsyn interaction on cytoskeleton modeling, axonal development and synaptic activity in neurons may be more complex as first thought [119, 140, 142]. Additional mechanisms, acting together in a vicious cycle, may explain how αsyn triggers tau aggregation and *vice versa*. Reciprocal promotion of phosphorylation is probably a key player, suggesting that kinases could be used as targets. GSK3β inhibition for instance was concomitantly able to decrease tau phosphorylation, αsyn accumulation and cell death in a cellular MPTP model [91]. In a P301L tau overexpressing mouse line, inhibition of tau hyperphosphorylation by treatment with a non-specific protein kinase inhibitor prevented motor impairments [99].

A mechanistic cross-seeding effect based on templating of a pathological β-sheet conformation is also highly suspected [151, 159–161]. Indeed, αsyn and tau can form co-oligomers that catalyze aggregation and finally lead to the formation of pure homofibrils [100, 158]. This resembles the prion self-propagation mechanism and this parallel is now commonly drawn in the literature, even if there is no evidence of human-to-human transmission for αsyn or tau [162, 163]. However, mutual misfolding is probably the first event that leads to αsyn and tau synergistic co-aggregation. The fact that αsyn has homology with the 14-3-3 co-chaperone protein, a known partner of tau and αsyn, and is able to substitute 14-3-3 co-chaperone activity on 14-3-3 targets supports this hypothesis [136, 137]. In this regard upregulation of chaperone proteins is another promising strategy that is presently being investigated to restore the normal conformation of αsyn and tau (Figure 4). Heat shock proteins (HSPs) increase the association of tau with microtubules and regulate tau degradation, ubiquitination and phosphorylation [164, 165]. Hsp70 preferentially binds to tau oligomers and restores anterograde fast axonal transport [166]. Our group has demonstrated that αsyn aggregation can be blocked by modulating different chaperones including Hsp27, Hsp70, Hsp90, torsinA and CHIP [167–175]. HSPs have been shown to positively act on αsyn or on tau independently, but they may also have a neuroprotective effect by restoring and regulating the normal interaction between both proteins [130, 132].

Observations suggest that αsyn being a trigger of tauopathy is more plausible than the opposite scenario. For instance, whereas the presence of tau only accelerated αsyn polymerization, co-incubation with αsyn was necessary to trigger tau aggregation *in vitro* [100]. Consistently, tau ablation failed to prevent neurotoxicity in the 6-OHDA or wt αsyn overexpressing mouse models [125]. Furthermore, pronounced tauopathy has been described in αsyn transgenic mice [100–102, 104, 105] and in PD patients harboring the A53T mutation [31, 33, 34], whereas no consistent αsyn pathology has been reported in tau transgenic mice or FTDP-17 T patients [66]. However, this is somewhat contradicted by the observation that αsyn pathology in AD is more pronounced than the tau pathology in PD [16, 22–24, 26–29, 37].

Nonetheless, even if some gray areas persist regarding the mechanisms and roles of the interaction between tau and αsyn, applications and future directions are already emerging. One future development is the identification of reliable biomarkers for efficient diagnosis of neurodegenerative disorders at the prodromal stage. In addition to other proteins, αsyn and tau are presently being developed as cerebrospinal fluid biomarkers for improved clinical diagnoses. Cerebrospinal fluid levels of Aβ, total and phospho-αsyn, and total and phospho-tau change differentially depending on the nature of the disease. Consequently, looking at the ratios between these proteins could enable clinicians to determine the risk of developing PD, AD, or a mixed disorder such as DLB and provide them the needed therapeutic window to start preventive and tailor-made treatments [176].

## Abbreviations

AD: Alzheimer’s disease
ALS: Amyotrophic lateral sclerosis
αsyn: Alpha-synuclein protein
DLB: Dementia with Lewy bodies
CBD: Corticobasal degeneration
FRET: Förster resonance energy transfer
FTDP-17 T: Frontotemporal dementia with parkinsonism linked to tau mutations on chromosome 17
GSK3β: Glycogen synthase kinase 3 beta
Hsps: Heat shock proteins
JNK: c-Jun N-terminal kinases
KO: Knock-out
LRRK2: Leucine reach repeat kinase 2
MAPK: Mitogen-activated protein kinase
*MAPT*: Tau gene
MPTP: 1-methyl-4-phenyl-1:2:3:6-tetrahydropyridine
MSA: Multiple system atrophy
PD: Parkinson’s disease
PDD: Parkinson’s disease with dementia
PiD: Pick’s disease
PKA: Protein kinase A
PSP: Progressive supranuclear palsy
*SNCA*: αsyn gene
SNP: Single nucleotide polymorphisms
wt: Wild-type.
